# Report to the Chicago Academy of Sciences

**Published:** 1866-05

**Authors:** 


### CHICAGO ACADEMY OF SCIENCES.—TRICHINÆ.

The following report, presented to a recent meeting of the Academy of Sciences, will be read with interest and profit. The character of the Committee is a full guarantee of the reliability of the facts presented in the Report. Let all who continue to eat pork be certain that it has been thoroughly cooked. —[Ed.]

Your Committee appointed at the last regular meeting to examine into the facts concerning the supposed existence of trichinæ in pork raised in this country, and the liability to disease which it may occasion, have the honor to make the following report:—

In preface it is proper to state that no reliable observations have ever yet been made in this country, with the view of deciding the question of the occurrence of trichinæ in our pork. Their existence here has been repeatedly denied, but the denial has never been based upon sufficient research. Your Committee became aware at the outset that the task imposed upon them was one far too arduous for their unassisted labors. The importance of the subject and the magnitude of the interests involved, indicated the necessity of an investigation of the most thorough character, and the propriety of accumulating the results of a large number of observations in order that the conclusions should be based upon data of such extent and variety that all causes of error might be eliminated.

To this end we have availed ourselves of the permission granted us at the time of the appointment of the Committee, and have added to the original number our colleague, Dr. Hosmer A. Johnson. We have also been fortunate in securing the aid of a number of professional gentlemen residing in our city, who have relieved us of much of the labor connected with the very extensive series of microscopic examinations of pork, necessary to the attainment of reliable and definite results. To these gentlemen, Drs. HOLLISTER, JEWELL, SHERMAN, NICKERSON, HAY, LYMAN, and NASON, we beg leave here to offer our acknowledgments for the assistance rendered.

*Its Character and History.*—Before presenting the results of our observations it may not be out of place to give a brief sketch of the character and history of the little helminth which is justly causing so much excitement among consumers of pork, a kind of animal food which is probably more extensively employed and more generally useful, not to say indispensable, than any other. Our own studies upon the structure and migrations of this helminth have indeed developed nothing new; but they have been sufficient to satisfy us of the accuracy of the most recent observations of the German naturalists, LEUCKART, and VIRCHOW in particular, who have devoted their attention to the subject. Trichina spiralis has been known for 30 years as an endo-parasite in the muscles of man, which could be bred in the muscles of some other mammals by feeding them with it. More recently it has been discovered to occur naturally in the muscles of swine. It is a minute, slender, and nearly transparent nematoid worm, scarcely 1-20th of an inch in length, even in its adult state, with a bluntly rounded posterior end, and an attenuated anterior extremity, at the tip of which the mouth is situated. Its internal structure, even when fully developed, is very simple. There is a straight alimentary canal, the æsophagial portion of which is enveloped in a glandular sheath; a yellowish globular organ at the middle, and a generative sac, nearly filling the posterior half of the body. In the encysted state in which the worm is most commonly observed, the latter organs are not fully developed.

*History of the Trichina.*—The life history of the trichina has been ascertained to be as follows:—

It is introduced in its encysted state into the stomach of man, or some other mammal, which may be susceptible to its ravages, and which may feed upon flesh infested with it. Very shortly the worms are freed from their capsules by the action of the digestive fluid and range freely in the stomach and intestines of the custodian. Their development proceeds rapidly and procreation takes place within four or five days after their ingestion. The males bear to the females the proportion of one-tenth only. The females are ovo-vivaparous, each giving birth to from 60 to 100 young and dying shortly thereafter. The young are extremely minute and thread-like, and remain for a short time lurking in the mucus of the lining membrane of the alimentary canal, where they cause great intestinal irritation, diarrhœa, and even death, if present in sufficient numbers. During the purging, many of the young trichinæ are swept from the intestinal canal with the fecal matters, in which they probably remain for some time alive, and it is supposed that swine sometimes derive their parasites from this source. After reaching a proper degree of size and strength, the young trichinæ begin to penetrate the walls of the intestines, and make their way toward their proper home, the voluntary muscles. They are now found in the glands of the mesentery and in the serous cavities. When numerous they cause great disorders in their passage and the animal infested, which has survived the previous intestinal irritation and its consequences, may now succumb from peritonæal inflammation.

*The Migratory State.*—In the course of their migration the trichinæ gradually assume the cork-screw form. They first reach the muscles immediately surrounding or traversing the cavity of the body, such as the diaphragm, lumbar, pharyngeal, intercostal, and abnormal muscles. Many doubtless take up therein their permanent abode, but it has been observed that they have a tendency, especially when very numerous, to push toward the extremities, or to pass between the ribs into the spinal muscles. This tendency to wide distribution is also shown in the fact that they are found more abundantly in the distal extremity of each muscle,—that farthest from the intestinal tract, in which situation their migration appears to have been stopped by the impervious tendon. In traversing the muscles, as far as has been observed, they do not penetrate the fibres, but wind their way between them. During this operation they cause in the affected man or animal great muscular pains and soreness, cramps, and even tetanic symptoms.

The migration of the young trichinæ occupies about four weeks. We have now traced them up to the point at which they become encysted. Of the causes which guide them in each individual case in the selection of their hibernaculum,—why some should encyst themselves close to the intestinal canal, while others proceed even to the extremities of the body, we know but little. It is probable, however, that this depends upon their supply of food and degree of development, and, that, after a proper condition is reached, a slight obstacle may arrest their course and cause them to encyst. Thus we have found them in every instance most abundant, not in the centre of masses of pure fibre, but in the immediate vicinity of some such obstacle as tendon, adipose tissue, or even the investing membrane (connecting tissue) of the fasciculi. It is the muscular fibre only that they affect, none having ever been found encysted in fat or the other tissues.

*The Encysted State.*—Having arrived at the terminus of its migration, the worm perforates the wall of the fibre selected, passes into it and flattens or tightens its coil in order to accommodate itself to the circumscribed space. Even then, however, the coil is so much larger than the diameter of the fibre as to dilate it at the point to two or three times its natural width. Immediately around its coil the trichina secrets a delicate membranous sac, and the enlarged cavity of the fibre becomes filled with a granular detritus, which is itself shortly enclosed by the action of the vital forces of the muscle, in a second envelope, the whole forming the fusiform cyst. This cyst gradually becomes calcareous by secretions from the surrounding parts and in time may become sufficiently cretified to be apparent from its whiteness to the naked eye. But it is only in man that these *calcareous* cysts have been observed,—hogs being generally killed long before time is afforded for the accumulation of sufficient lime. When the trychinæ have all become encysted, the acute pains usually cease in the subject. The amount of inconvenience afterwards experienced depends entirely upon the number of the fibres rendered inert by the presence of the parasites. This number is often sufficiently great to produce partial paralysis. Cases have occurred for instance, in which the use of the fingers has been entirely lost while other members retained their power. The parasites have a tendency to crowd into certain parts and leave others unaffected.

*The Torpid State.*—The trichina has now reached its quiescent or torpid state, in which it must remain during the life of its custodian, unless dislodged by accident. It feeds no longer, yet its development goes slowly on, until it has reached the condition of puberty. From this time it must remain unaltered, awaiting the chances of its freedom, dependant upon the ingestion of the flesh containing it by some animal carnavorously inclined. Then the cycle goes on as already detailed. These helminths can breed but once in the body of one and the same animal.

*Origin of the Trichina.*—With regard to the origin of the trichina little or nothing is known. It has been thus far found to occur naturally only in man, the hog, and the cat. All statements of its natural occurrence in other mammals seem to have been founded on error, although some of them may be easily infested by special feeding. Above all, reports of its occurrence in earth-worms and other cold-blooded animals are to be entirely discredited. These latter are indeed infested with tilariæ, and other nematoid worms, somewhat resembling trichinæ; but the practised zoologist easily distinguishes between them. The occurrence of trichinæ in man and in the cat is easily accounted for,—both have probably eaten of infested pork. But how shall we account for the existence of the worm in the hog? We can scarcely account for all cases of trichiasis in these animals upon the supposition that they have eaten of the flesh of man, cats, or of other hogs, although the small proportion of hogs infested might lend some color of probability to this supposition. The question remains for further investigation.

*Period of Existence.*—As before mentioned, trichinæ have been known for 30 years to exist in the muscles of man. But it is only within a very few years that their deadily character, when introduced into the system in considerable numbers, has been fully understood, and has attracted the attention of the medical profession. The terrible catastrophies of Hedersleben and Hettstadt, at the former of which 100 and at the latter 83 persons were the victims of a meal of trichinous pork, with many minor disasters, have not failed to excite in Europe the deepest interest and the most careful study of the ravages of the flesh worm. To such an extent, in fact, have these disasters alarmed the people that in many parts of Europe no pork is allowed to be sold without having previously undergone microscopic examination.

*Tenacity of Life.*—The deadly character of the trichinæ depends upon their great tenacity of life. Total decomposition of the muscle containing them will not effect their vitality, and, in cooking, their destruction can only be accomplished by the application of a heat above 150 degrees Fahrenheit.

*Analysis.*—Your Committee have conceived that the object for which they were appointed is twofold:—first, to ascertain whether trichinæ actually exists in the hogs of this country and in those of the northwest in particular; and, secondly, should they exist, to determine the extent of the danger thereby incurred, and to ascertain the best means of averting it. For the attainment of the first-mentioned object they have, with the assistance of the gentlemen named at the head of this report, procured and examined portions of muscle taken from 1,394 hogs in the different packing houses and butcher stalls of our city. The results of these examinations have been engrossed in the tables herewith presented. The first of these shows the number of specimens, and in most cases the names of the muscles examined by each observer, with the number of trichinous specimens found by each. The second gives various data concerning the 28 trichinous specimens found, which are numbered in the order of their discovery, and are preserved in the cabinet of the academy:—

| Observer. | Pharyngeals. | Diaphragm. | Abdominals. | Intercostals. | Lumbars. | Spinals. | Various. | Total. | Trichinous specimens. |
|---|---|---|---|---|---|---|---|---|---|
| Dr. Johnson | 1 | 7 | ---- | ---- | 15 | 224 | 74 | 321 | 13 |
| Dr. Sherman | 17 | 9 | 30 | 37 | ---- | 35 | 2 | 130 | 2 |
| Dr. Nickerson | ---- | ---- | 10 | 9 | ---- | 107 | 124 | 250 | 3 |
| Dr. Jewell | ---- | ---- | ---- | ---- | ---- | ---- | 212 | 212 | 3 |
| Dr. Hollister | ---- | ---- | ---- | ---- | ---- | ---- | 70 | 70 | 2 |
| Dr. Nelson | ---- | ---- | ---- | ---- | ---- | ---- | 50 | 50 | 1 |
| Drs. Blaney & Hay | 4 | 9 | 8 | ---- | ---- | 10 | 3 | 34 | ---- |
| Dr. Nason | ---- | ---- | 9 | 7 | ---- | 31 | ---- | 47 | ---- |
| Dr. Andrews | ---- | ---- | ---- | ---- | ---- | ---- | 96 | 96 | 1 |
| Dr. Lyman | ---- | ---- | ---- | ---- | ---- | ---- | 90 | 90 | 1 |
| Dr. Stimpson | 6 | 8 | ---- | ---- | 16 | 61 | 3 | 94 | 2 |
| Total | ---- | ---- | ---- | ---- | ---- | ---- | ---- | 1394 | 28 |

| No. | Observer. | Muscle. | No. to a cubic in. |
|---|---|---|---|
| 1. | Dr. Johnson | Spinal | 350 |
| 2. | Dr. Johnson | Spinal | 300 |
| 3. | Dr. Johnson | Spinal | 200 |
| 4. | Dr. Johnson | Intercostal | 100 |
| 5. | Dr. Johnson | Intercostal | 500 |
| 6. | Dr. Johnson | Spinal | 600 |
| 7. | Dr. Johnson | Abdominal | 3,000 |
| 8. | Dr. Johnson | Spinal | 500 |
| 9. | Dr. Johnson | Spinal | 1,000 |
| 10. | Dr. Stimpson | Spinal | 18,000 |
| 11. | Dr. Johnson | Spinal | 15,000 |
| 12. | Dr. Johnson | Spinal | 300 |
| 13. | Dr. Johnson | Spinal | 300 |
| 14. | Dr. Johnson | Spinal | 300 |
| 15. | Dr. Stimpson | Spinal | 400 |
| 16. | Dr. Nickerson | Unknown | 48 |
| 17. | Dr. Nickerson | Unknown | 80 |
| 18. | Dr. Nickerson | Unknown | 192 |
| 19. | Dr. Sherman | Unknown | 3,000 |
| 20. | Dr. Sherman | Pharyngcel | 6,000 |
| 21. | Dr. Andrews | Abdominal | 2,000 |
| 22. | Dr. Nelson | Spinal | 2,000 |
| 23. | Dr. Lyman | Unknown | 16,000 |
| 24. | Dr. Hollister | Unknown | 500 |
| 25. | Dr. Hollister | Unknown | 500 |

| | | | |
|---|---|---|---|
| 26. | Dr. Jewell | Unknown | 2,000 |
| 27. | Dr. Jewell | Unknown | 250 |
| 28. | Dr. Jewell | Unknown | 500 |

*The Proportion of Diseased Hogs.*—By these tables it will be perceived that we have found trichinæ in the muscles of 28 hogs out of the 1,394 examined. We may therefore conclude that in the hogs brought to Chicago 1 in 50 is affected with trichiniasis in a greater or less degree. We must confess our surprise at arriving at this result, which indicates with little doubt the startling fact that trichiniasis in pork is even more common in this country than in Germany, where it has caused so much suffering and death. For instance, in the city of Brunswick, where a most careful inspection of 19,747 hogs was made in the years 1864-5, only two were found to contain trichinæ in their muscles, the proportion being 1-10,000 against 1-50, as before stated in our country.

*Our Immunity.*—The comparative immunity from the disease which our own people have enjoyed, undoubtedly results from the habit of cooking meat before eating it, while in Germany it is eaten raw by the poorer classes on account of the high price of fuel.

*Variation in Number.*—It will be also observed by consulting the tables that the specimens examined show great variation in the number of worms infesting them. We have given, indeed, only an approximation to the number existing in a cubic inch in each specimen of muscle, but this approximation is sufficiently near the truth for our present purposes. Our method has been to count the trichinæ occurring in several different portions of muscle, each a cubic tenth of an inch in size, and to multiply the average number by 1,000, to find the number to a cubic inch. By this method we find that only three of our specimens (Nos. 10, 11, and 23) contain over 10,000 to the cubic inch and are therefore as densely infested with the worm as the pork which has occasioned the disasters in Germany. The remaining 25 are infested in a comparatively slight degree, viz.:—from 48 to 6,000 to the cubic inch. The specimen most thickly infested contains 18,000 to the cubic inch, and we have calculated that a person eating an ordinary meal of this pork in a raw state would speedily become a victim to the ravages of not less than 1,000,000 of young trichinæ. In certain cases of death from trichiniasis, the number found in the muscles of man has been 2,000,000.

With regard to the muscles of the hog most liable to be infested, we have to state that our determinations do not accord with those of European observers, inasmuch as more than half of our trichinous specimens have been taken from the spinal muscles. In Europe the microscopic inspectors of pork are directed to examine nine different sets of muscles, viz.:—Those of the diaphragm, tenderloin, shoulder, front and back of neck, intercostals, extensors of fore-arm, flexors of the leg and the muscles of the larynx. In this list the spinal muscles are not even mentioned, although their examination with us has proved the most prolific in results, even after making allowance for the fact that we have searched these muscles more than any others. Our experience has led us to believe that a much smaller number of examinations than are required by the European rules will suffice for the detection of trichinæ in dangerous numbers in pork. The occurrence of a number so small as to escape observation, in a search of two or three of the muscles most liable to be infested, we do not conceive to be dangerous when we consider that the greater proportion of the worms must be killed even by the ordinary method of cooking.

*Defence against its Ravages.*—Now that the existence of trichinæ in our pork has been established beyond a doubt, it will be proper for us to point out all known means of defence against its ravages.

First, with regard to the rearing of hogs. These animals undoubtedly become infested through the eating of flesh of some kind, since no trichinæ nor germs of trichinæ have ever been found in any vegetable food. A strict attention to the feeding of hogs and their confinement in pens where no animal food is accessible in an infallible preventive against trichiniasis in them. Such management is all the more necessary, since European authorities agree that it is impossible to diagnose the disease in the animal from external appearances, and no culpability can therefore attach to the farmer for selling hogs which prove to be affected with trichinæ.

In regard to pork, the origin of which is doubtful, the use of the microscope is primarily indicated. With this instrument only can we ascertain with certainty whether the muscles of the hog are free from the parasite. The general use of this instrument is, however, impracticable, unless a system of microscopic inspection be adopted here as in Europe at the great packing establishments. But we have in our power much more simple means of insuring safety in the consumption of pork. It is simply necessary to cook it thoroughly so that every portion of the meat shall have experienced a temperature of at least 160 degrees Farenheit. We cannot insist too strongly on this point. Again by properly salting and smoking the meat for at least ten days, the trichinæ should they exist, will certainly be killed. Simple dessication of the meat, if continued for a period of sufficient length, will also kill them. They will never be found alive in old hams, for instance. On the other hand, mere pickling appears to have very little effect upon these worms.

*Existence before Discovery.*—Trichinæ have doubtless always existed in the muscles of the hog, although probably not to the same extent as at present, and trichiniasis in man may have existed to a considerable extent in this country before its nature and cause became known. Some of the members of your Committee can recall cases of obscure diseases which have come to their knowledge in past years, which may have been owing to the presence of trichinæ.

*Economical Aspects.*—Having now fully exposed the exact extent of the danger from trichinous pork, to which our people are liable, and stated the means of avoiding it, we will proceed to close our report with a few remarks upon the economical aspects of the subject. A panic has been produced in the mind of our public by the news which has reached us from Germany concerning the disasters which have occasionally followed the consumption of pork in a raw state. The excitement has, with little doubt, been fostered by interested persons, for speculative purposes, until people have come to imagine there is a danger in eating pork of any kind,—a danger all the more terrible because hidden, little understood, and undiscoverable by ordinary means. All this excitement has occurred, before a single instance of the occurrence of trichinæ in American hogs has been, as far as we are aware, authentically reported. It has therefore become necessary that the subject should be thoroughly investigated, in order that the people, by familiarity of the danger and confidence in their understanding of its character, may not be the pray of superstitious fears. The panic which now prevails is unfounded in reason, senseless, and greatly injurious. We do not allude to the commercial aspects of the question, a matter of small moment compared with the great importance of pork as the kind of meat diet upon which nine-tenths of our agricultural population, north and south, mainly depend. In our view it would be folly to discard this kind of meat from our list of articles of food, when all possibility of injury attending its use may be avoided by the most simple means. Let the people but understand that only 1 hog in 48 contains trichinæ at all, that only 1 in 300 contains them in sufficient numbers to cause considerable danger, and that even in these cases the worms are rendered innocuous by proper smoking, drying, or cooking,—and we imagine that few sensible persons will refuse pork as food if it suits their convenience to use it.

(Signed) E. Andrews, M.D.,
J. V. Z. Blaney, M.D.,
Hosmer A. Johnson, M.D.,
William Stimpson. M.D.,
*Secretary.*

